# Study of the Operational Safety of a Vascular Interventional Surgical Robotic System

**DOI:** 10.3390/mi9030119

**Published:** 2018-03-08

**Authors:** Jian Guo, Xiaoliang Jin, Shuxiang Guo

**Affiliations:** 1 Key Laboratory for Control Theory and Application in Complicated Systems and Biomedical Robot Laboratory, Tianjin University of Technology, Tianjin 300384, China; 163126319@stud.tjut.edu.cn; 2 School of Life Science, Key Laboratory of Convergence Medical Engineering System and Healthcare Technology, The Ministry of Industry and Information Technology, The Institute of Advanced Biomedical Engineering System, Beijing Institute of Technology, Beijing 100081, China; 3 Intelligent Mechanical Systems Engineering Department, Kagawa University, Takamatsu 761-0396, Japan

**Keywords:** vascular interventional surgery (VIS), safety early warning, master–slave tracking error, displacement error compensation algorithm, blood vessels, force feedback

## Abstract

This paper proposes an operation safety early warning system based on LabView (2014, National Instruments Corporation, Austin, TX, USA) for vascular interventional surgery (VIS) robotic system. The system not only provides intuitive visual feedback information for the surgeon, but also has a safety early warning function. It is well known that blood vessels differ in their ability to withstand stress in different age groups, therefore, the operation safety early warning system based on LabView has a vascular safety threshold function that changes in real-time, which can be oriented to different age groups of patients and a broader applicable scope. In addition, the tracing performance of the slave manipulator to the master manipulator is also an important index for operation safety. Therefore, we also transformed the slave manipulator and integrated the displacement error compensation algorithm in order to improve the tracking ability of the slave manipulator to the master manipulator and reduce master–slave tracking errors. We performed experiments “in vitro” to validate the proposed system. According to previous studies, 0.12 N is the maximum force when the blood vessel wall has been penetrated. Experimental results showed that the proposed operation safety early warning system based on LabView combined with operating force feedback can effectively avoid excessive collisions between the surgical catheter and vessel wall to avoid vascular puncture. The force feedback error of the proposed system is maintained between ±20 mN, which is within the allowable safety range and meets our design requirements. Therefore, the proposed system can ensure the safety of surgery.

## 1. Introduction

Cardiovascular and cerebrovascular diseases have become one of three major causes of human death, posing a serious threat to human health. Even in developed countries, cardiovascular disease is still the leading cause of death, accounting for 34% of deaths each year [1].

Vascular interventional surgery is a common, minimally-invasive surgery to treat heart-related pathology, which involves the complex skill required to manipulate a flexible catheter or a guide wire within human vascular vessels to access a target and carry out operations [2]. It has some advantages, such as short hospital stays, less incisions and less recovery time [3]. Nonetheless, the operation has obvious disadvantages: the surgery needs to be carried out under the guidance of medical imaging equipment, causing damage to the surgeon’s body [4,5]. In addition, due to the high risks involved, the surgeon must be highly skilled and specialized. In order to solve this problem, our team has developed a master–slave interventional surgery robotic system. It can separate doctors and patients, so that the doctor can be away from harmful radiation, and can also reduce the dependence on experts who can perform this complex operation, allowing more doctors to master the vascular interventional technology and accelerate patients’ access to surgical treatment [6].

In recent years, the use of medical and surgery robotic systems has become a popular topic of study. Most medical and surgical robotic systems include both the master manipulator and the slave manipulator [7]. The main challenges regarding minimally-invasive surgeries performed with the assistance of medical–surgical catheter robots are measuring the interaction force between the slave manipulator of surgery robotic system and the environment, and the lack of haptic sensation provided to the human operator [8]. However, to solve the vascular interventional surgery problems mentioned above, the combination of robot technology and vascular interventional technology is very important [9,10,11]. In addition, efficient surgical robotic systems should be used to help surgeons manipulate catheterization in a safe space [12]. The physiological tremors and miss operations of a surgeon can be filtered out through the system, increasing the success of the surgery [13]. Many research groups around the world are committed to the development of surgery robotic systems. Guiatni M. and Riboulet V. et al. have presented a new interface for minimally-invasive surgery training that incorporates novel broadband sensory modalities—including visual, force, and thermal technology—into the evolution of the next generation of surgical robotics and simulators [14]. Talasaz et al. have presented the relevance of force feedback (presented visually as well as directly) during tactile sensing (only presented visually) for tumor localization using an experimental setup close to one that could be applied to real robotics-assisted, minimally-invasive surgeries [15]. The design concept of a human-operator-centered haptic interface was firstly introduced by Yin et al. [8,16]. A new, compact and sterilizable tele-robotic system with three degrees of freedom was proposed, which allowed the interventionalist to use conventional steerable catheters [17]. An overview of the vascular interventional robot was reported. Typically, a skilled surgeon is needed to guide a catheter or endoscope. No existing surgery robotic system can match a surgeon’s skill at inserting and rotating a catheter to train unskilled surgeons [18]. Most of the research is focused on the realization of force feedback and the design of master–slave structures, there is little research on the safety of the surgery robotic system. Therefore, for operational safety issues, our team transformed the slave manipulator structure and reduced the master and slave tracking error; we also designed an operation safety early warning system based on LabView for a vascular intervention surgery robotic system to enhance the visual feedback effect and ensure the safety of the surgical robot system.

In this paper, we proposed an operation safety early warning system. This system not only provides intuitive visual feedback information for the operator, but also has a safety early warning function. We also transformed the slave manipulator and integrated the displacement error compensation algorithm in order to improve the tracking ability of the slave manipulator to the master manipulator. In addition, the proposed operation safety early warning system combined with operating force feedback can effectively avoid excessive collisions between the surgical catheter and the vessel wall, bringing the force feedback error within the allowable range (0–0.12 N) and meeting our design requirements.

## 2. Robotic System Description

**Figure 1:**
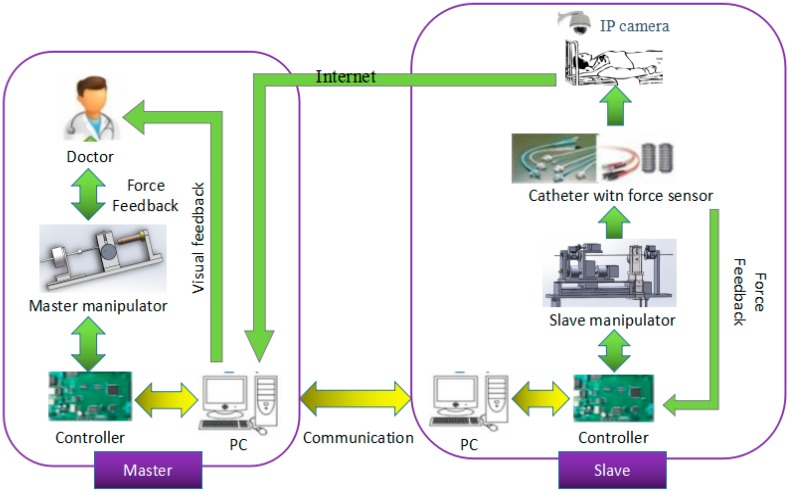
The structure of the master–slave robotic system for vascular intervention surgery.

The surgical robotic system is a closed loop system, composed of the master side and the slave side; the structure is shown in Figure 1. The doctor operates the master manipulator on the master side, the master controller (STM32F103ZE-EK, STMicroelectronics, Geneva, Switzerland) sends the collected information to the master personal computer (PC), the master PC sends the information to the slave PC through the user datagram protocol (UDP) communication, and then sends the information to the slave controller (STM32F103ZE-EK, STMicroelectronics, Geneva, Switzerland), thereby driving the slave side to make the appropriate movement to complete the vascular intervention surgery. During operation, a fiber pressure sensor (OPP-M40, Opsens, Quebec City, QC, Canada) is used to measure the contact force when the tip of the surgical catheter (Cordis brite tip GC, 8F, Kaneka Corporation, Tokyo, Japan) collides with the vessel wall. A load cell (TU-UJ5N, TEAC, Tokyo, Japan) is used to measure the resistance of the surgical catheter when the surgical catheter is inserted into the human blood vessel. The coil of the master manipulator is energized and then moves the operation catheter to perform the magnetic flux movement to produce electromagnetic force as a feedback force that can be felt by the doctor’s hand and enable the realization of force feedback.

### 2.1. Master Manipulator

**Figure 2:**
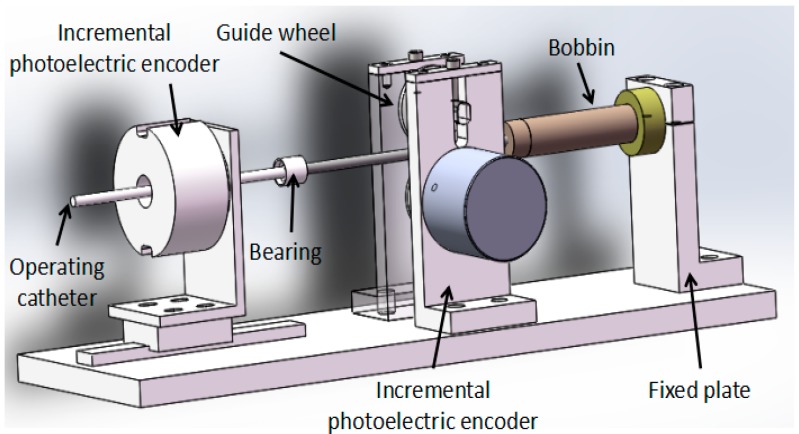
The structure of the master manipulator.

The master manipulator consists of two parts, which are the motion information acquisition unit and the force feedback unit; the structure is shown in Figure 2. The motion information includes axial displacement information and radial rotation information. It uses an incremental photoelectric encoder (ZSP3806-2500BM, Jinan Ke Sheng Automation Technology Co. Ltd., Jinan, China) to detect motion information that is mainly composed of a grating disk and photoelectric detection device. Using a 2500-line incremental photoelectric encoder, it is connected to a HCTL-2016 (Agilent Technologies, Santa Clara, CA, USA) quadrature decoder chip to form four times the frequency. It then goes through 10,000 lines in each lap, with an incremental photoelectric encoder solid shaft circumference of 100 mm, so that the incremental photoelectric encoder for each step is the distance of the catheter movement, or 0.01 mm, and the axial measurement accuracy is 0.01 mm. The axial rotation angle of the incremental photoelectric encoder is transformed into a straight line displacement, and the measurement accuracy is higher than that of the linear displacement sensor.

When the coil is energized and the operation catheter is moved by the operator, the bobbin follows the movement. The permanent magnet then forms a uniform magnetic field. The electric coil generates electromagnetic force in a uniform magnetic field. The electromagnetic force as a feedback force can be transmitted to doctor’s hand through the operation catheter.

### 2.2. Slave Manipulator

**Figure 3:**
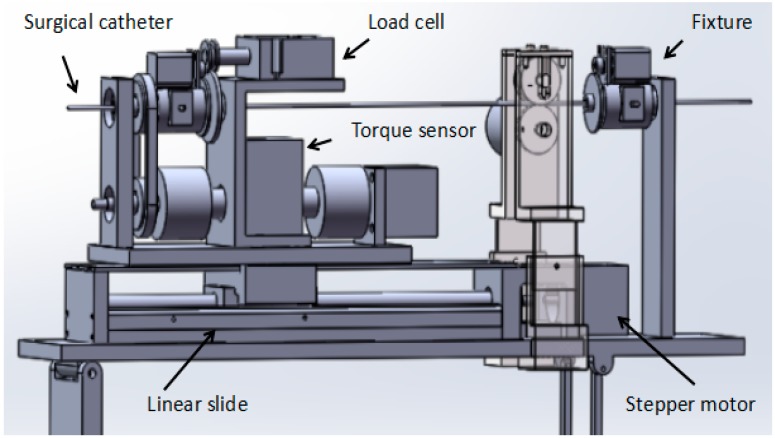
The novel structure of the slave manipulator.

The novel slave manipulator mainly includes a surgical catheter motion-driven unit, a motion information detection unit and a force information detection unit, as shown in Figure 3.

The axial motion driven unit with high-precision stepper motor (AR24SAKD-N10-1, Tianjin yat bochi technology Co. Ltd., Tianjin, China) drives a linear slide to control the surgical catheter forward and backward. The stepper motor can continue to run even in the event of a sudden change in load and it can start and stop with a faster response frequency. The noise of the operation is relatively low. The stepper motor used in this system is a harmonic deceleration motor with a resolution of 1000 P/R. When the motor speed reduction ratio is 100, the angle of one pulse of the stepper motor is 0.0036 degrees. Since the motor outputs a rotary motion, it is necessary to connect a linear slide to convert it into a linear motion. The linear slide consists of two main components: the ball screw and the slider. This is an extension of the ball screw that allows the radial rotation action, which is converted into an axial forward and backward movement, with very low frictional resistance, realizing high-precision linear motion. The linear slide in this system has a stroke of 400 mm. The radial motion-driven unit also uses a stepper motor, with a resolution of 1600 P/R, and the angle of one pulse of the stepper motor is 0.225 degrees. The motor is rotary-driven through the timing belt and pulley to achieve surgical catheter synchronous movement [19].

The motion information detection unit mainly comprises an incremental photoelectric encoder and the structure for fixing the incremental photoelectric encoder. The incremental photoelectric encoder is used to measure the displacement information of the surgical catheter. The displacement information is returned to the master to achieve displacement feedback.

The force information detection unit mainly comprises a load cell (TU-UJ5N, TEAC, Tokyo, Japan) and a torque sensor (TRD-N1000B, Tianjin runda zhongke instrument Co. Ltd., Tianjin, China). The load cell is used to measure the resistance when the surgical catheter is in the process of inserting human blood vessels, and sends resistance information back to the master side to achieve force feedback. The load cell in the system was made by the Japanese TEAC company, and measures the axial thrust by measuring the shaft. The output signal of the sensor is passed through the amplifier circuit method and transmitted to the analog-to-digital (AD) conversion module to achieve data acquisition. The maximum thrust force that can be detected is +5 N and the maximum pulling force is −5 N.

## 3. Methods

### 3.1. Operation Safety Early Warning System Design

**Figure 4:**
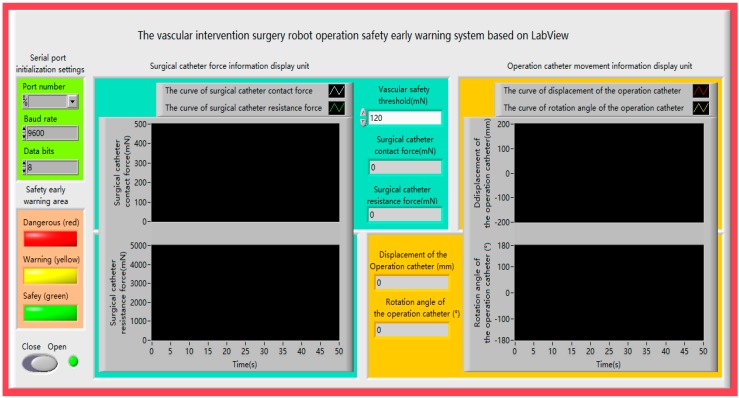
The operation safety early warning system.

The operation safety early warning system consists of four parts: serial port initialization setting, safety early warning area, surgical catheter force information display unit and operation catheter movement information display unit, as shown in Figure 4. Description of the operation safety early warning system functionalities as shown in Table 1.

**Figure 5:**
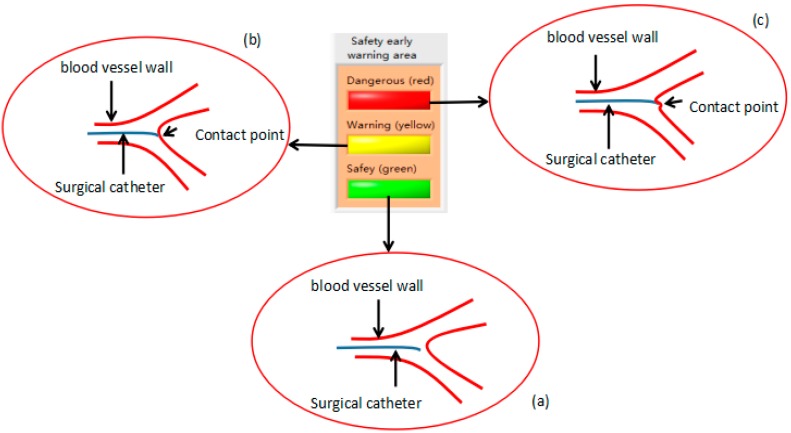
(**a**) The contact force of the tip of the surgical catheter is less than the vascular safety threshold. (**b**) The contact force of the tip of the surgical catheter is equal to the vascular safety threshold. (**c**) The contact force of the tip of the surgical catheter exceeds the vascular safety threshold.

We used the fiber pressure sensor (OPP-M40, Opsens, Quebec City, QC, Canada), which is mainly composed of a fiber optic probe and signal demodulator, a probe diameter of 0.25 mm, and a resolution of 0.5 mmHg. The data measured by the sensor will be transmitted to the PC through the RS232 serial port. As shown in Figure 5a, the safety lamp (light on: green, light off: dark green) of the system will be lit when the contact force of the tip of the surgical catheter is less than the vascular safety threshold (other lamps are light off). As shown in Figure 5b, the warning lamp (light on: yellow, light off: dark green) of the system will be lit when the contact force of the tip of the surgical catheter is equal to the vascular safety threshold (other lamps are light off), under the circumstances, the doctor should be careful when operating. As shown in Figure 5c, the danger lamp (light on: red, light off: dark green) of the system will be lit when the contact force of the tip of the surgical catheter is greater than the vascular safety threshold (other lamps are light off), at this time, the doctor must stop the current operation. The advantage of the safety early warning system is that it prevents the puncture of blood vessels by the surgical catheter and avoids unnecessary injury to patients.

### 3.2. Control Algorithm

**Figure 6:**
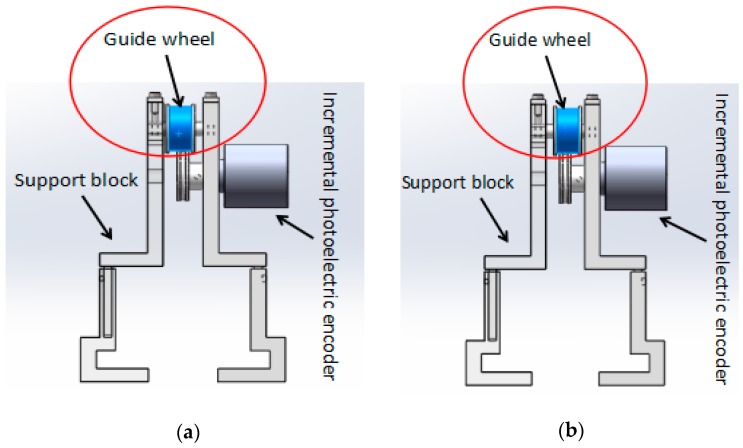
The structure of the surgical catheter displacement error compensation (**a**) before the surgical catheter is rotated; (**b**) after the surgical catheter is rotated.

In the master–slave, minimally-invasive robotic system for vascular intervention surgery, error is inevitable while the slave manipulator is tracking the master manipulator. This error also reduces the safety of the surgery. In order to reduce the error, we designed a new structure for surgical catheter displacement error compensation. As shown in Figure 6, the structure is mainly composed of a support block that can be adjusted in height, an incremental photoelectric encoder (ZSP3806-2500BM, Jinan Ke Sheng Automation Technology Co. Ltd., Jinan, China) and a guide wheel. The support block, which can adjust the height, ensures the surgical catheter in a horizontal state and reduces the friction between the surgical catheter and the guide wheel. In addition, the structure is portable, can be removed and is easy to operate. The incremental photoelectric encoder can detect the axial displacement of the surgical catheter and send it to the master controller (STM32F103ZE-EK, STMicroelectronics, Geneva, Switzerland). The guide wheel is used to fix the surgical catheter. The rotation of the surgical catheter is achieved by the friction between the surgical catheter and the guide wheel. Figure 6a shows the view before the surgical catheter rotates, Figure 6b shows the view after the surgical catheter rotates.

**Figure 7:**
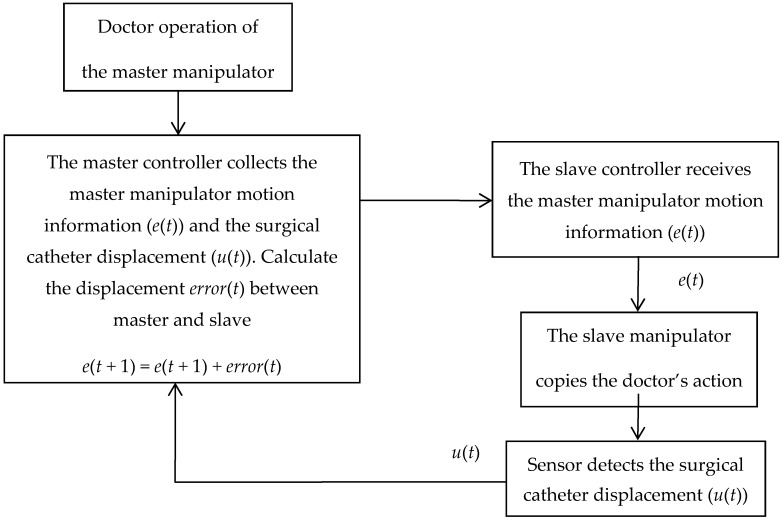
The principle of the surgical catheter displacement error compensation algorithm.

The principle of the surgical catheter displacement error compensation algorithm is shown in Figure 7. The slave controller (STM32F103ZE-EK, STMicroelectronics, Geneva, Switzerland) receives the master manipulator motion information (*e(t)*) via the serial port and drives the slave manipulator to copy the doctor’s action. The sensor detects the surgical catheter displacement (*u(t)*) and sends it back to the master controller (STM32F103ZE-EK, STMicroelectronics, Geneva, Switzerland) to achieve motion information feedback. The master controller compares the displacement value of the slave surgical catheter with the displacement value of the master operating catheter through the program. When the displacement value of the slave surgical catheter is equal to the displacement value of the master operating catheter, the controller will directly exit the program and then wait to receive the next data. When the displacement value of the slave surgical catheter is not equal to the amount of displacement of the master operating catheter, the *error*(*t*) between the master and slave displacement will be calculated, and the error will then be sent to the slave controller again to achieve error compensation, and then exit the program, as shown in Equations (1) and (2).

(1) error(t)=e(t)−u(t)

(2) e(t+1)=e(t+1)+error(t)

### 3.3. Remote Operation Interactive System

**Figure 8:**
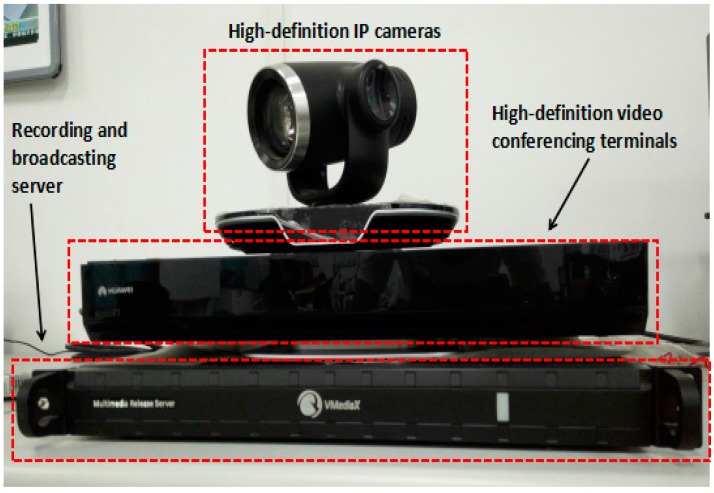
Remote operation interactive system.

Remote operation interactive system (TE40, Huawei Technologies Co. Ltd., Shenzhen, China), shown in Figure 8, mainly includes three parts: high-definition internet protocol (IP) cameras, high-definition video conferencing terminals, and a recording and broadcasting server.

High-definition IP cameras: support 8× optical zoom support 1080 P 50/60 fps, 1080 i50/60, 1080 P 25/30, 720 P 50/60 fps video output; support 2.38 million pixel 1/2.8 inch complementary metal oxide semiconductor (CMOS) imaging chip; horizontal viewing angle can reach 72 degrees, the maximum vertical angle of 44.5 degrees with an external wide-angle lens.

High-definition video conferencing terminal: Automatically obtain the IP address of the network, when two high-definition video conferencing terminals work together, one of high-definition video conferencing terminals calls the other high-definition video conferencing terminal through the network. They establish connections and achieve surgical scene interaction.

Recording and broadcasting server: Using an Internet Explorer (IE) browser and entering the video server IP address, one can log in to the video recording window and record the whole surgical procedure.

## 4. Experimental and Results

### 4.1. Experimental Setup

**Figure 9:**
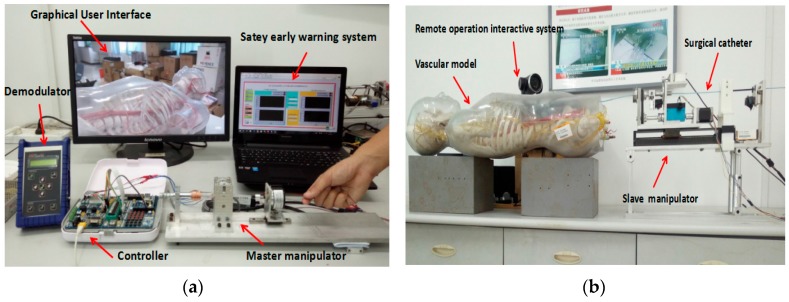
The master–slave, minimally-invasive robotic system for vascular intervention (**a**) The master (doctor) side; (**b**) The slave (patient) side.

As shown in Figure 9a, the master side includes the operation safety early warning system, which programmed in LabVIEW 2014 (National Instruments, Austin, TX, USA), a graphical user interface (GUI), a fiber pressure sensor demodulator (OPP-M40, Opsens, Quebec City, QC, Canada), a controller (STM32F103ZE-EK, STMicroelectronics, Geneva, Switzerland) and the master manipulator. As shown in Figure 9b, the slave side includes a vascular model (KAD/A10005, Shanghai standard and poor laboratory equipment Co. Ltd., Shanghai, China), a remote operation interactive system (TE40, Huawei Technologies Co. Ltd., Shenzhen, China), a controller (STM32F103ZE-EK, STMicroelectronics, Geneva, Switzerland) and the slave manipulator.

**Figure 10:**
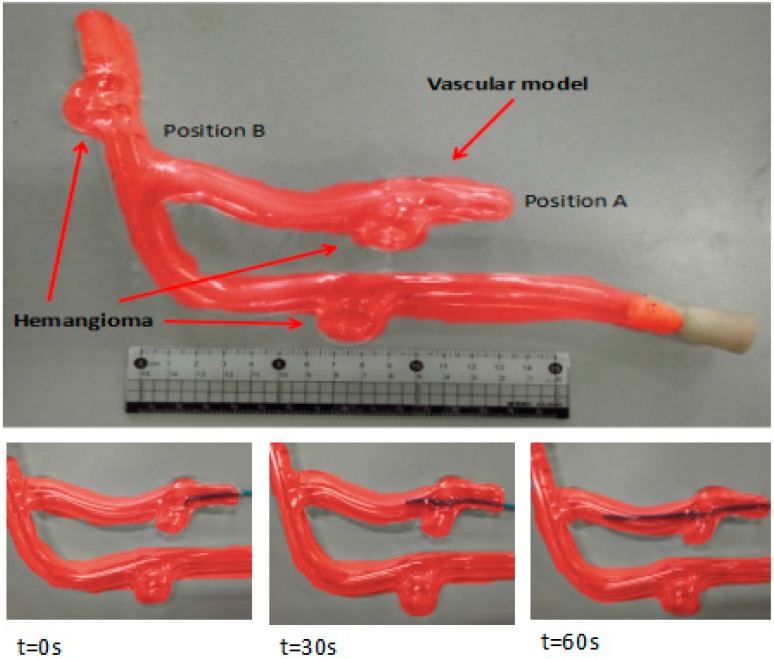
Experimental blood vessel model.

The experimental process was as follows: we injected physiological saline into the experimental vascular model instead of real blood and set the vascular safety threshold to 0.12 N—0.12 N is the force when the blood vessel wall has been penetrated [20,21]. A staff member, under the guidance of a doctor, operates the master manipulator on the master side, and the surgical catheter on the slave side moves from position A to position B, as shown in Figure 10. Surgical catheter force information and operation catheter motion information are fed back to the operation safety early warning system through the serial port (potr:COM3, Baud rate: 115,200, Data bit:8). The remote operation interactive system monitors the operation scenes from the slave (patient) side and feed back to the GUI on the master side.

### 4.2. The Calibration between the Electromagnetic Induction Damping Force and Current

**Figure 11:**
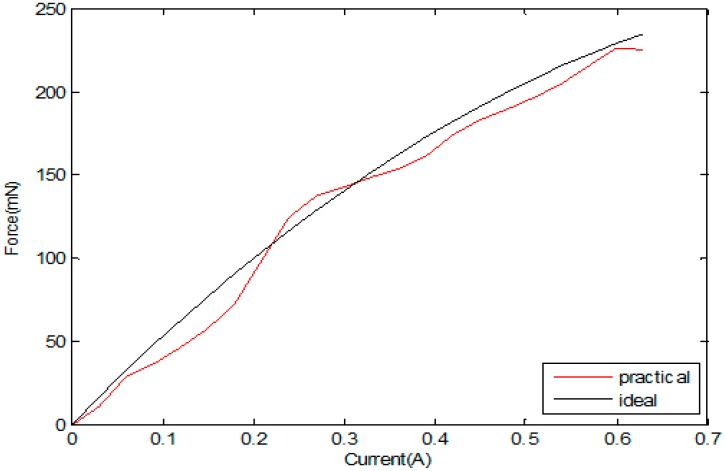
The calibration experiment results for the force and current.

In the calibration test, we can obtain the relation of the current and damping force. Figure 11 shows the calibration experiment result of force and current. Until the coil is energized, a magnetic coil is formed due to the influence of the permanent magnet field. As shown in Figure 11, with the increase in the coil current, the damping force also increases. When the voltage-controlled current source adjusts the current to 0.6 A, the re-adjustment current no longer changes, and the damping force reaches the maximum of 241 mN. Figure 11 shows the results of the experiment on the magnetic force of the measured values in the permitted range. Based on the correlation data between the input current and the magnetic force, the fitting curve equation was established with MATLAB (2010, MathWorks, Inc., Natick, MA, USA), where F is the electromagnetic force and I is the input current [22].

According to the measured value, using MATLAB for the second fitting, the input current and electromagnetic induction of the mathematical relationship between the equations was calculated, as shown in Equation (3) based on Equation (4).

(3) $$F=-290I^{2}+556I$$

Because the current supplied to the electromagnetic induction damper coil cannot directly change the current through the controller (STM32F103ZE-EK, STMicroelectronics, Geneva, Switzerland), our team has designed a voltage–current conversion circuit to change the magnitude of the coil current in the electromagnetic induction damper and the electromagnetic induction damping force. In this circuit, the resistance *R*_1_ = 2 kΩ, and the resistance values *R*_2_ = 1 kΩ, and *R*_4_ = 1 Ω, *I* represents the current through the coil on the bobbin, *V_in_* represents the control voltage, as shown in Equation (4). The control voltage is output from the digital-to-analog (DA) converter of the master controller (STM32F103ZE-EK, STMicroelectronics, Geneva, Switzerland) to the voltage-controlled, constant-current source circuit, and the current in the coil on the bobbin is changed by the controller.

(4) $$I=\frac{V_{\mathrm{in}}\times R_{2}}{R_{4}\times (R_{1}+R_{2})}$$

### 4.3. Operation Safety Early Warning System Evaluation

**Figure 12:**
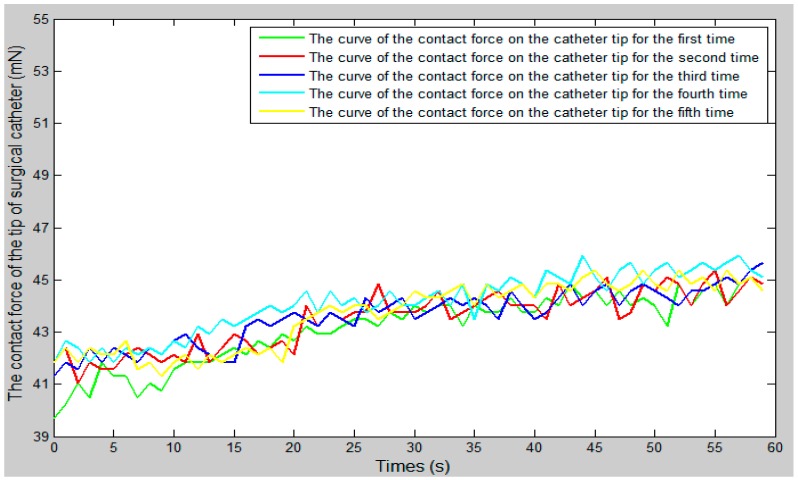
The curve of the contact force of the surgical catheter.

**Figure 13:**
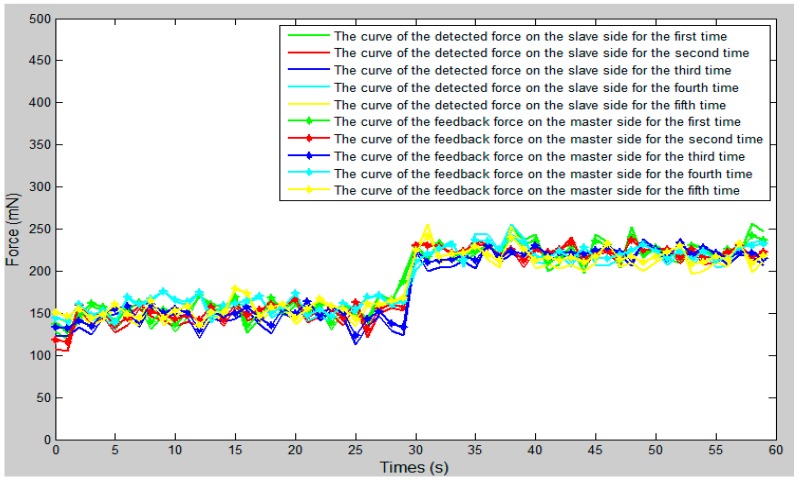
The comparison curve between the slave detected force with the master feedback force.

**Figure 14:**
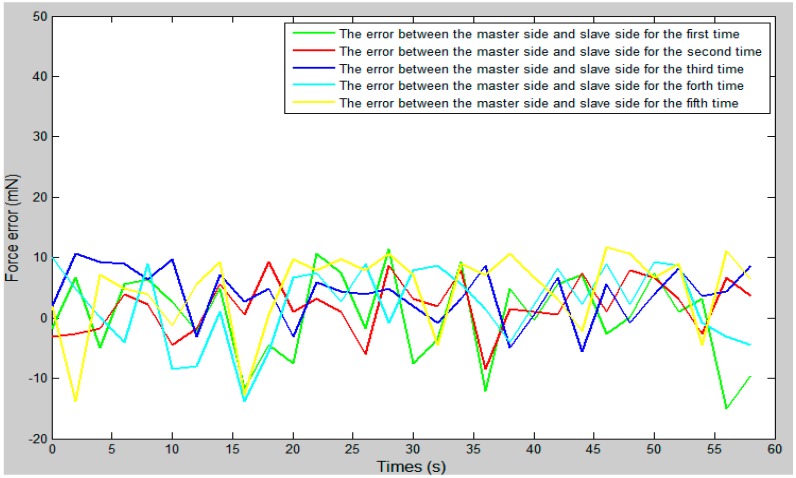
The error curve between the slave detected force with the master feedback force.

We set the vascular safety threshold to 0.12 N and performed five experiments, pushing the surgical catheter from position A to position B in the experimental blood vessel model, as shown in Figure 10. The force information of the surgical catheter and the movement information of the operation catheter were transmitted through the serial port to the operation safety early warning system on the master PC. According to the status of the safety early warning area, a staff member under the guidance of a doctor operated the master manipulator (push–pull and rotate) and made sure the contact force of the tip of the surgical catheter was in the safety range (0–0.12 N). The experimental results as shown in Figure 12, Figure 13 and Figure 14, through the analysis of the experimental data, verify the effectiveness of the operation safety early warning system.

As shown in Figure 12, the whole curve shows a slow upward trend. The main reason for this is that with the operation of the simulation surgery, the surgical catheter will get deeper and deeper into the blood vessels, and the corresponding pressure is going to become greater and greater. Therefore, the contact force of the surgical catheter will also show an upward trend. When the surgical catheter is in contact with the blood vessel wall, one should adjust the motion state of the operation catheter, move the catheter from the slave side through the bend of the vessel, and continue to push forward the catheter.

As shown in Figure 13, between 27 s and 30 s, the surgical catheter begins to make contact with the vessel wall, as due to the friction of the vascular wall and the surgical catheter, the resistance of the surgical catheter is increased. Between 30 s and 60 s, the fluctuation range of the curve was smaller and remained stable because of the friction between the wall of the catheter and the wall of the vessel. As shown in Figure 14, the force feedback error of the proposed system is maintained between ±20 mN. Compared with previous studies [23], the error value is within the allowable range and met our design requirements.

### 4.4. Control Algorithm Evaluation

**Figure 15:**
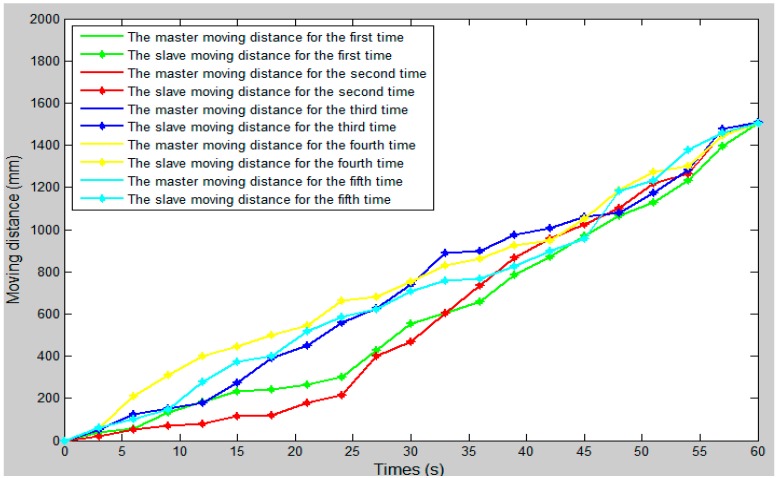
The comparison curve between the slave moving distance and the master moving distance.

**Figure 16:**
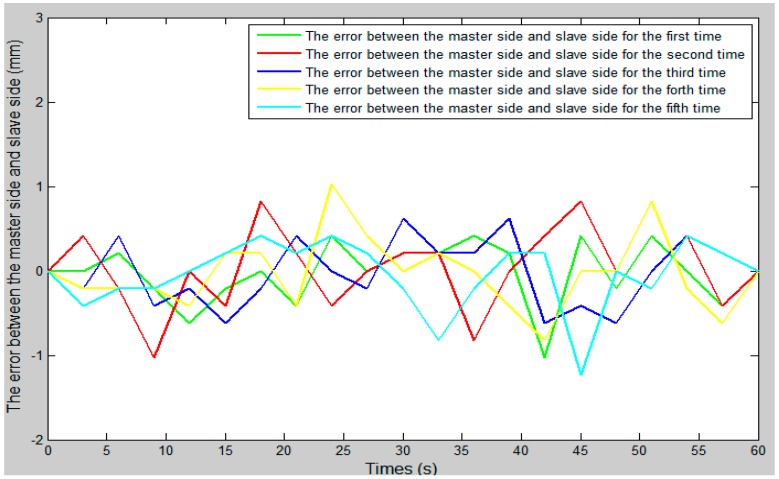
The error curve between the slave moving distance and the master moving distance.

**Figure 17:**
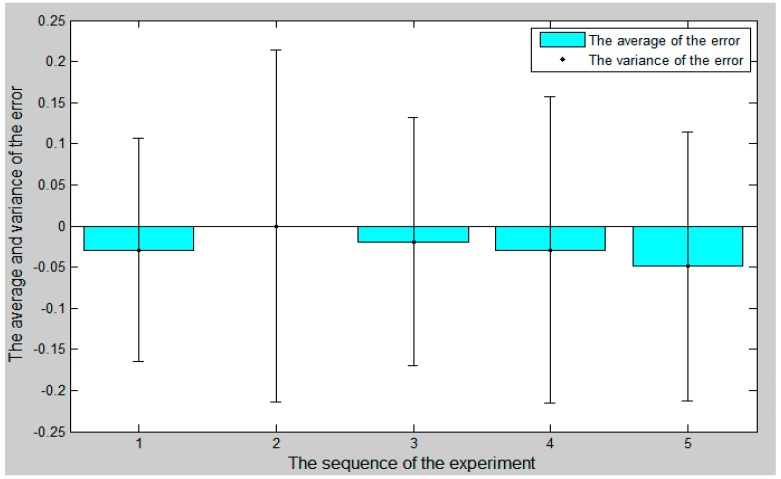
The master–slave axial displacement sequence of the experiment.

In the whole experiment, the blood vessel model was 150 mm from position A to position B, as in the experimental blood vessel model shown in Figure 10. The comparison curve between the slave moving distance and the master moving distance is shown in Figure 15. The error curve between the slave moving distance and the master moving distance is shown in Figure 16, and the master–slave axial displacement sequence of the experiment is shown in Figure 17. According to Figure 16, which shows the error curve of the axial displacement, the error in the experiment is kept in the range of plus or minus 1 mm, which meets experimental requirements. Compared with previous generations of tracking experiments of master–slave displacement, the performance of the slave manipulator is improved [23]. Therefore, the operation safety of the master–slave, minimally-invasive robotic system for vascular interventions is improved.

**Figure 18:**
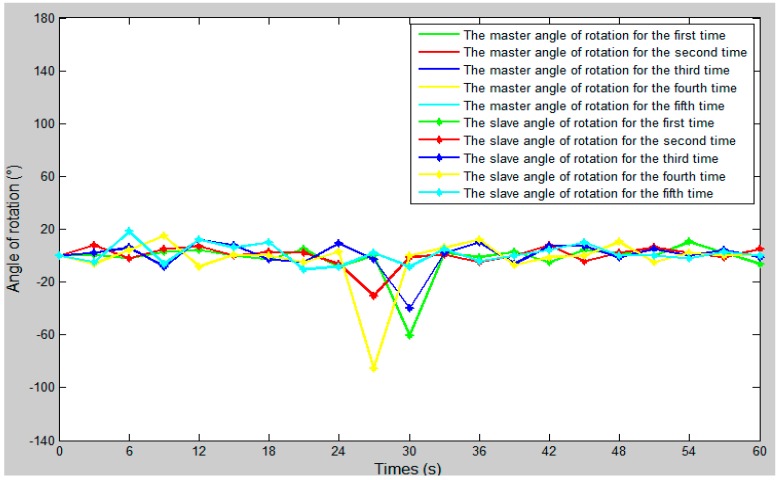
The comparison curve between the slave angle of rotation and the master angle of rotation.

**Figure 19:**
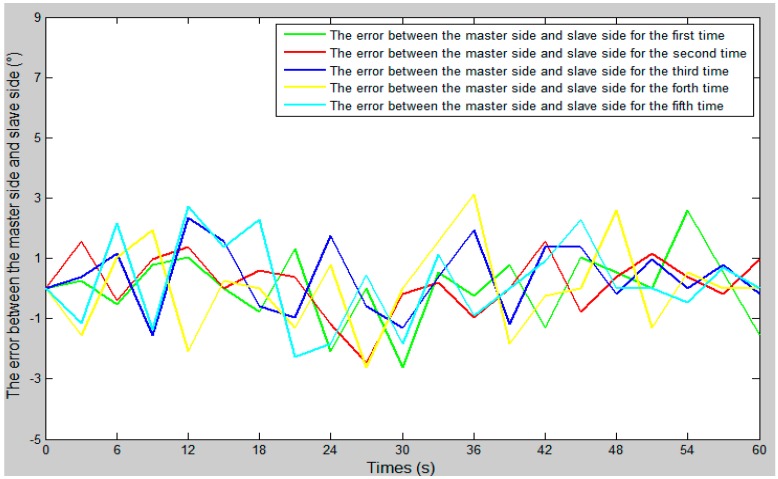
The error curve between the slave angle of rotation and the master angle of rotation

**Figure 20:**
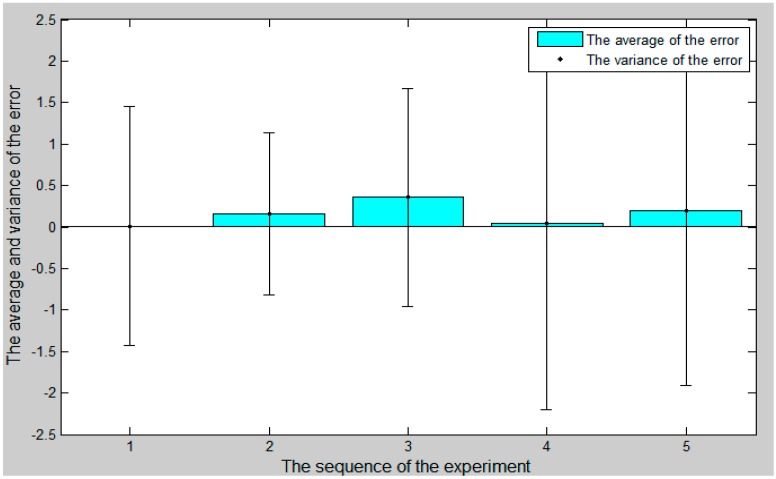
The master–slave radial rotation angle sequence of the experiment

The comparison curve between the slave angle of rotation and the master angle of rotation is shown in Figure 18. The error curve between the slave angle of rotation and the master angle of rotation is shown in Figure 19, and the master–slave radial rotation angle sequence of the experiment is shown in Figure 20. According to Figure 18, between 27 s and 30 s, when the surgical catheter reaches the bend of the vascular model, we properly rotate the master operation catheter, and then control the rotation of the surgical catheter so that the surgical catheter can smoothly pass through the vessel bend and ensure surgical safety.

## 5. Discussion

In this paper, we proposed an operation safety early warning system based on LabVIEW. We also transformed the slave manipulator and integrated the displacement error compensation algorithm in order to improve the tracking ability of the slave manipulator to the master manipulator and reduce the master–slave tracking error.

We used a fiber pressure sensor (OPP-M40, Opsens, Quebec City, QC, Canada) to measure the contact force between the catheter tip and the vessel wall, which is mainly composed of a fiber optic probe and signal demodulator, a probe diameter of 0.25 mm, and a resolution of 0.5 mmHg. It has the following characteristics: (1) Small catheter dimensions. (2) The measurement was not affected by electronic surgery and nuclear magnetic resonance (NMR). (3) There is a higher fidelity than a fluid-filled sensor. (4) Better performance than other pressure sensors (no humidity drift).

We used STM32 (STM32F103ZE-EK, STMicroelectronics, Geneva, Switzerland) as a controller. STM32 has the following characteristics: (1) Use the advanced architecture of the cortex-m3 kernel. (2) Excellent real-time performance. (3) Outstanding power consumption control. (4) Outstanding and innovative peripherals. (5) Higher degree of integration and easy to develop. (6) The CPU frequency is 72 MHz.

**Figure 21:**
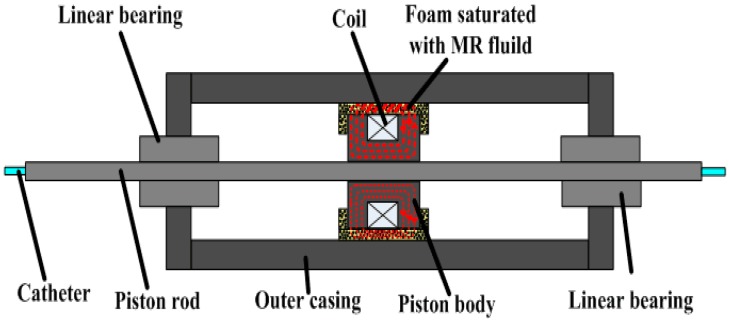
Magnetorheological (MR) fluid damper assembly.

At present, many research teams are conducting research on force feedback. For example, in reference [24], they designed a pneumatically actuated master robot (haptic device) with strain-gauge-based force sensing that was configured to operate the slave from within the scanner room during imaging. In reference [25], Hu et al. designed a natural haptic user interface for a non-link-coupled mechanical surgical robot. The haptic device must be portable to avoid constraining the user’s body. They achieved this objective considering that the only mechanical structure in the optical-motion-capture master is the forceps-like hand interface. All of the optical markers were mounted on this interface. Both grasp force feedback and push–pull force feedback are provided to the hand interface to signal normal stress (F_n_) and translational shearing stress (F_t_) to the user’s hand. In reference [26], in order to give a haptic force feedback while the catheter is remotely advanced, Marcelli E, Bortolani B, Cercenelli L, et al. inserted a vibration motor in the push-button box in the proximity of the mock handle. The vibration was activated when the alarm or stop threshold was exceeded, thus providing a reaction force back to the operator’s hand. An acoustic alarm was also added to the vibrational haptic feedback. Each research team has a different approach to realizing force feedback. As far as our team is concerned, we are also constantly improving the force feedback system. The novel master manipulator adopts the electromagnetic induction principle to realize the force feedback, and we made a detailed introduction in Section 2.1 and Section 4.2. Our previous research [23] used the magnetorheological (MR) fluid principle to realize the force feedback, the schematic is shown in Figure 21. When the piston moves, the MR fluid in the foam produces a force which is called the shear stress. The foam is like a reservoir of MR fluid and absorbs the maximum amount of MR fluid. The piston has a number of turns of coil to produce the required magnetic field. As the current in the coil is increased to its maximum value, the magnetic field in the MR sponge is increased and the shear stress is produced. As a result, it resists the shearing motion of the piston.

In the in vitro experiment, we injected physiological saline into the experimental vascular model instead of real blood and set the vascular safety threshold to 0.12 N—0.12 N is the force when the blood vessel wall has been penetrated [20,21]. A staff member under the guidance of a doctor operated the master manipulator on the master side, and the surgical catheter on the slave side moves from position A to position B, as in the experimental blood vessel model shown in Figure 10. Surgical catheter force information and operation catheter motion information are fed back to the operation safety early warning system through the serial port (port number: COM3, baud rate: 115200, data bit: 8). The remote operation interactive system (TE40, Huawei Technologies Co. Ltd.) monitors the operation scenes from the slave (patient) side and feed back to the GUI on the master side.

The experimental results showed that the operation safety early warning system combined with operating force feedback can effectively prevent the phenomenon of catheter puncture. Under the successful completion of the experiment, the contact force of the tip of the surgical catheter was maintained below the safety threshold to ensure the safety of the operation. According to previous studies, 0.12 N is the force when the blood vessel wall has been penetrated [20,21]. Compared to previous research by our team [23], the algorithm for surgical catheter displacement error compensation improved the motion tracking ability of the slave manipulator to the master manipulator and compensated the movement error of the surgical catheter and improved the operation safety of the surgical robot. In addition, the force feedback error of the system is maintained between ±20 mN. This indicated that the error was within the allowable range and met our design requirements. Based on the above three points, the designed system can meet our design requirements.

## 6. Conclusions

In this paper, we proposed an operation safety early warning system. The system not only provides intuitive visual feedback information for the master operator, but also has a safety early warning function. In addition, we also transformed the slave manipulator and integrated the displacement error compensation algorithm, which improved the slave manipulator’s ability to track the master manipulator and the safety of surgical robot operation.

Besides, we performed experiments “in vitro” to validate the proposed system. According to previous studies, 0.12 N is the force when the blood vessel wall has been penetrated. The experimental results show that the proposed operation safety early warning system combined with operating force feedback can effectively avoid excessive collisions between the surgical catheter and the vessel wall to avoid vascular puncture. The force feedback error of the proposed system is maintained between ±20 mN. This indicated that the error is within the allowable range and met our design requirements. Therefore, the proposed system can ensure the safety of the surgery.

In the future, we will carry out experiments “in vivo” using the developed robotic catheter system.

## Figures and Tables

**Table 1:** Description of the operation safety early warning system functionalities.

Block	Component	Function Description
Serial port initialization settings	Port number Baud rate Data bits	Keep data transmission
Surgical catheter force information display unit	Vascular safety threshold Waveform display Numerical display	Sets the safety threshold Provides visual feedback
Operation catheter movement information display unit	Waveform display Numerical display	Provides visual feedback
Safety early warning area	Dangerous led (red) Warning led (yellow) Safety led (green)	Indicates stop current operation Indicates operation warning, be careful Indicates operation safety
Start/stop commands	Start softkey Stop softkey	Starts the system Stops the system
